# Association Between Microplastic Exposure and Cognitive Function Decline

**DOI:** 10.1002/alz70860_100679

**Published:** 2025-12-23

**Authors:** Pukovisa Prawiroharjo, Anyelir Nielya Mutiara Putri, Noryanto Ikhromi, Elizabeth Divina, Rani Permata, Talitha Najmillah Sabtiari, Alditya Fakhri, Alya Ayu Tazkia, Viola Aprillia, Dinda Nisrina

**Affiliations:** ^1^ Department of Neurology, Faculty of Medicine, Universitas Indonesia, Jakarta, Indonesia, Central Jakarta, Jakarta, Indonesia; ^2^ Dr. Cipto Mangunkusumo Public Hospital, Central Jakarta, DKI Jakarta, Indonesia

## Abstract

**Background:**

Microplastics (MPs) are environmental pollutants that have increased alongside the widespread use of plastic. Exposure to MPs through daily consumption can impact health, including cognitive function, via mechanisms of toxicity, oxidative stress, and inflammation. This study aimed to evaluate the relationship between MP exposure patterns, MP concentrations in biological fluids (blood, urine, and feces), and their effects on cognitive function.

**Method:**

The study was conducted in two phases. The first phase involved an online survey of 562 respondents in the Greater Jakarta area to assess plastic consumption patterns, health complaints, and awareness and attitudes toward MPs. The second phase recruited 67 respondents from the highest (decile 10) and lowest (decile 1) exposure groups for biological sample collection (blood, urine, and feces). Analyses were performed to determine the relationship between MP exposure levels, MP concentrations in biological fluids, and cognitive function, measured using the Montreal Cognitive Assessment Indonesia (MoCA‐Ina).

**Result:**

Polyethylene terephthalate (PET) and polypropylene (PP) microplastics were detected in respondents' blood, urine, and feces at varying concentrations. A significant association was observed between MP exposure levels and MP concentrations in biological fluids. Higher MP concentrations were linked to cognitive decline, particularly among respondents with the highest MP levels. Risk perception regarding MPs was higher among respondents with greater awareness and knowledge, while attitudes toward MPs correlated with plastic consumption patterns

**Conclusion:**

This study demonstrates a significant relationship between MP exposure and health impacts, particularly cognitive decline. These findings underscore the urgency of reducing plastic use, raising public awareness, and implementing policies for plastic waste management to mitigate health risks associated with MPs

